# Rectal atresia and rectal stenosis: the ARM-Net Consortium experience

**DOI:** 10.1007/s00383-023-05518-7

**Published:** 2023-07-28

**Authors:** Cunera M. C. de Beaufort, Ramon R. Gorter, Barbara D. Iacobelli, Paola Midrio, Cornelius E. J. Sloots, Inbal Samuk, Iris A. L. M. van Rooij, Gabriele Lisi, for the ARM-Net Consortium, Ivo de Blaauw, Francesco Fascetti-Leon, Araceli García Vázquez, Wilfried Krois, Martin Lacher, Ernesto Leva, Eberhard Schmiedeke, Nagoud Schukfeh, Michael Stanton

**Affiliations:** 1 Department of Pediatric Surgery, Emma Children’s Hospital, Amsterdam UMC, Location University of Amsterdam, Meibergdreef 9, 1105 AZ Amsterdam, The Netherlands; 2 Amsterdam Gastroenterology Endocrinology Metabolism, Meibergdreef 9, Amsterdam, The Netherlands; 3 Amsterdam UMC, Amsterdam Reproduction and Development Research Institute, University of Amsterdam, Meibergdreef 9, Amsterdam, The Netherlands; 4 Neonatal Surgery Unit, Clinical Area of Fetal, Neonatal and Cardiological Sciences, Bambino Gesù Childrens Hospital, IRCCS, Rome, Italy; 5 Department of Pediatric Surgery, Cà Foncello Hospital, Treviso, Italy; 6 Department of Pediatric Surgery, Sophia Children’s Hospital, Erasmus MC, Rotterdam, The Netherlands; 7 Department of Pediatric and Adolescent Surgery, Schneider Children’s Medical Center of Israel, Sackler School of Medicine, Tel Aviv University, Petah Tikva, Israel; 8 Department for Health Evidence, Radboud University Medical Center, Nijmegen, The Netherlands; 9 Department of Pediatric Surgery, Spirito Santo Hospital, Pescara, G. d’Annunzio University, Chieti-Pescara, Italy

**Keywords:** Rectal atresia, Rectal stenosis, Anorectal malformations, Constipation, ARM-Net Consortium

## Abstract

**Purpose:**

To assess the number, characteristics, and functional short-, and midterm outcomes of patients with rectal atresia (RA) and stenosis (RS) in the ARM-Net registry.

**Methods:**

Patients with RA/RS were retrieved from the ARM-Net registry. Patient characteristics, associated anomalies, surgical approach, and functional bowel outcomes at 1 and 5-year follow-up were assessed.

**Results:**

The ARM-Net registry included 2619 patients, of whom 36 (1.3%) had RA/RS. Median age at follow-up was 7.0 years (IQR 2.3–9.0). Twenty-three patients (63.9%, RA n = 13, RS n = 10) had additional anomalies. PSARP was the most performed reconstructive surgery for both RA (n = 9) and RS (n = 6) patients. At 1-year follow-up, 11/24 patients with known data (45.8%, RA n = 5, RS n = 6) were constipated, of whom 9 required stool softeners and/or laxatives. At 5-year follow-up, 8/9 patients with known data (88.9%, RA n = 4, RS n = 4) were constipated, all requiring laxatives and/or enema.

**Conclusion:**

RA and RS are rare types of ARM, representing 1.3% of patients in the ARM-Net registry. Additional anomalies were present in majority of patients. Different surgical approaches were performed as reconstructive treatment, with constipation occurring in 46% and 89% of the patients at 1 and 5-year follow-up. However, accurate evaluation of long-term functional outcomes remains challenging.

**Supplementary Information:**

The online version contains supplementary material available at 10.1007/s00383-023-05518-7.

## Introduction

Rectal atresia (RA) and rectal stenosis (RS) are both rare/regional variants of anorectal malformation (ARM), occurring in 1–2% of neonates with ARM [1, 2]. RA/RS are often diagnosed late, potentially due to the normal appearing anus that is located appropriately in the midline, and within the sphincter mechanism [3]. As depicted by Sharma and Gupta, RA/RS can be subdivided into 5 types: type I: RS: (A) intramural, (B) web with a hole; type II: RA with a septal defect; type III: RA with a fibrous cord between two atretic ends; type IV: RA with a gap; type V: multiple: (A) RA with stenosis, (B) multiple RA, and (C) thickened Houston’s valves and/or multiple RS [4]. However, this classification is not yet universally accepted since rectal stenosis is often present in patients with specific features such as pre-sacral mass and/or Currarino syndrome (as is anal stenosis). However, patients with RS should be assessed and approached differently compared to anal stenosis, since they are considered to be different entities. As with other types of ARM, additional anomalies might be present in patients with RA/RS. Therefore, screening for these subsequent anomalies might be also of importance in patients with RA/RS [5].

Due to the rare nature of the subtypes of RA/RS, little is known about this patient population and the optimal technique for reconstructive treatment. Because RA and RS being two distinct congenital ARM, differences might be present in terms of surgical repair. Moreover, in nearly all patients with RA, initially a diverting colostomy is performed, followed by reconstructive surgery, but in some patients with RS, only dilatations are performed. Different techniques have been described for the definitive surgical correction of RA/RS such as pull-through procedures, transanal excision, posterior sagittal anorectoplasty (PSARP), and magnamosis [6–8]. The chosen approach might differ between types of RA/RS due to distinct disease morphology, clinical presentation, and the surgeon’s preference. Many of the described treatment strategies are invasive surgical procedures (e.g., PSARP, pull-through), and little is known about their mid- and long-term postoperative outcomes in this specific patient group [9]. As all patients with ARM, also patients with RA/RS might be prone to continence problems such as soiling or constipation in the long-term [10]. However, given the normal position of the atretic or stenotic rectum within the sphincter complex, patients with RA/RS might be candidate for good bowel control unless negative prognostic factors are present (e.g., sacral anomaly, spinal dysraphism, poor sacral ratio, and presence of syndromic anomalies) and any surgical procedure should optimize the prognosis for bowel control in the long-term [11].

Given the rarity of RA/RS, literature is scarce and mainly monocentric case reports and case series on specific aspects of the most appropriated treatment strategy are available. Therefore, the aim of this ARM-Net Consortium study is to assess the number and characteristics of patients with RA/RS, and their functional short- (1 year), and mid-term outcomes (5 years) reported in the ARM-Net registry.

## Methods

### Study design and patient population

This was a retrospective cohort study, designed in accordance with the Strengthening the Reporting of Observational Studies in Epidemiology (STROBE) guidelines [12]. Data were retrieved from the ARM-Net registry. This registry enrolls children aged under 3 years with ARM, born between 2008 and 2022, and treated in one of the ARM-Net Consortium participating centers. All patients with RA/RS in the ARM-Net registry were considered eligible for inclusion. Patients born with other types of ARM, including anal stenosis, were excluded.

### Ethics

According to the ARM-Net Consortium regulations, before entering patients into the ARM-Net registry, patient data is de-identified and pseudo anonymized, protecting patient privacy and meeting local ethical requirements varying within each center and country.

### Data extraction

Data on patient characteristics, type of ARM (i.e. RA or RS), (non-)syndromic anomalies (e.g., VACTERL-association, Currarino syndrome), the presence of a pre-sacral mass, additional anomalies (i.e. vertebral, cardiac, renal, genital, and limb anomalies), additional imaging studies (i.e. renal and spinal ultrasound (US), and vertebral x-ray), treatment strategy (i.e. dilatations, ostomy, and reconstructive intervention), postoperative complications, and 1-year follow-up outcome measures were extracted from the registry on March 1st 2023. In case data on patient characteristics, additional anomalies, and 1-year follow-up was missing, the surgeons who entered the patient into the ARM-Net registry were contacted through e-mail with the request of completing the file. In addition, since 5-year follow-up data was not registered in the ARM-Net registry, these data were also collected through email sent to the treating surgeon.

### Definitions

Patients were classified according to their type of RA/RS as reported in the ARM-Net registry, and if possible, additional classification was done according to the classification as suggested by Sharma and Gupta [4]. Radiology reports in the registry were scored on the presence of additional brain, cardiac, renal, vertebral, skeletal and spinal cord anomalies. No imaging was repeated. Functional outcomes at 5-year follow-up were reported according to the Rintala score [10, 13].

### Outcomes

Primary outcome was the number and characteristics of patients with RA/RS reported in the ARM-Net registry.

Secondary outcomes were the type of reconstructive treatment for RA/RS, and the functional 1- and 5-year bowel outcomes after intervention.

### Statistical analyses

Statistical analysis was conducted using IBM SPSS Statistics for Windows, Version 28 (IBM Corp., Armonk, N.Y., USA). Descriptive statistics were used for analysis of baseline characteristics. These were reported as proportions and percentages for binary or categorical variables, and as mean with standard deviation (SD) or as median with interquartile range (IQR) for continuous variables as appropriate. Univariable logistic regression analyses were performed to investigate associations between sex, ARM type, the presence of syndromes, and the presence of additional anomalies. Additionally, all variables used in the univariable analysis were selected for multivariable logistic regression analyses. Outcomes were reported as odds ratio (OR) with corresponding 95% confidence interval (95% CI). A p-value of < 0.05 was considered statistically significant. Missing or unknown data were described.

## Results

### Participants

In total, 2619 patients with ARM were identified in the ARM-Net registry, of whom 36 (1.3%) had RA (n = 18) or RS (n = 18). Median age at analysis was 7.0 years (IQR 2.3–9.0). Syndromic anomalies were identified in 4 patients (22.2%) with RA (Down syndrome n = 3, Currarino syndrome n = 1), and 6 patients (33.3%) with RS (Johanson-Blizzard syndrome n = 1, Currarino syndrome n = 5. One patient (2.8%) was part of twins. During the study period, 2 patients (5.6%) deceased (at 5 months and unknown age), both due to cardiac failure.

### Additional anomalies

Additional anomalies were identified in 23 patients (63.9%, RA n = 13, RS n = 10, of whom 9 had a single, and 12 had multiple additional anomalies. No differences were identified between the number of additional anomalies in patients with RS versus RA (see Table 1) (i.e. brain p = 0.15, cardiac p = 0.14, skeletal p = 0.48, spinal cord p = 0.67, renal p = 0.37, genital anomalies p = 0.15, and pre-sacral mass p = 0.15). Syndromes were independently associated with the presence of additional anomalies (OR 11.8, 95% CI 1.06–131.2, p = 0.05). In addition, no associations could be demonstrated between sex or subtype of ARM, and the presence of additional anomalies (Table 2).

**Table 1:** Baseline characteristics

	Rectal stenosis	Rectal atresia	Total
	n (%)	n (%)	n*(%)
Sex			
*Male*	16 (88.9)	10 (55.6)	26 (72.2)
*Female*	2 (11.1)	8 (44.4)	10 (27.8)
Treatment			
*Ostomy*	6 (33.3)	16 (88.9)	22 (73.3)
*Reconstructive intervention*	13 (72.2)	15 (83.3)	28 (93.3)
Syndromes	6 (33.3)	4 (22.2)	10 (27.8)
Additional anomaly			
*Single*	4 (22.2)	5 (27.8)	9 (25.0)
*Multiple*	6 (33.3)	8 (44.4)	12 (33.3)
Additional anomaly			
*Brain*	0 (0.0)	2 (11.1)	2 (5.6)
*Cardiac*	3 (16.7)	7 (38.9)	10 (27.8)
*Skeletal*	7 (38.9)	5 (27.8)	12 (33.3)
*Spinal cord*	3 (16.7)	4 (22.2)	7 (19.4)
*Renal*	2 (11.1)	4 (22.2)	6 (16.7)
*Genital*	0 (0.0)	2 (11.1)	2 (5.6)
*Pre-sacral mass*	2 (11.1)	0 (0.0)	2 (5.6)
Total patients	18 (50.0)	18 (50.0)	36 (100.0)
	Median (IQR)	Median (IQR)	Median (IQR)
Age			
*At analysis (years)*	7.0 (2.5–9.0)	9.5 (2.0–10.0)	7.0 (2.3–9.0)
*At reconstructive surgery (months)*	5.0 (2.0–6.25)	5.0 (2.0–6.0)	5.0 (2.0–6.0)

*n* number. *IQR* inter quartile range. *Numbers and percentages are of total known data, excluding unknown or missing data

**Table 2:** Uni- and multivariable logistic regression for the association between sex, type of ARM, the presence of syndromes and the presence of additional anomalies*

	Univariable		Multivariable	
	OR (95% CI)	p-value	OR (95% CI)	p-value
*Type of ARM*				
Rectal stenosis	Ref		Ref	
Rectal atresia	0.48 (0.12–1.93)	0.30	0.27 (0.05–1.59)	0.15
*Sex*				
Female	Ref		Ref	
Male	1.46 (0.31–6.98)	0.64	0.56 (0.08–3.40)	0.56
*Syndrome*				
Not present	Ref		Ref	
Present	7.71 (0.85–70.0)	0.07	11.8 (1.06–131.2)	**0.05**

Bold in univariable and multivariable analysis indicates statistical significance (p < 0.05). *Additional anomalies include any anomaly in brain, cardiac, skeletal, spinal cord, renal, genital, and a pre-sacral mass

### Treatment strategies

Twenty-two patients (73.3%, RA n = 16, RS n = 6) were initially treated with an ostomy, of whom 8 (36.4%) developed postoperative complications (i.e. wound dehiscence (n = 2), wound infection (n = 2), inverted stomas (n = 1), parastomal omentum prolapse (n = 1), stenosis (n = 1), and retraction requiring redo surgery (n = 1)). Data on reconstructive intervention was available for 30 patients (83.3%). In total, 28/30 patients (93.3%) underwent reconstructive intervention, including surgical (n = 26) and non-surgical intervention such as dilatations (n = 2) (see Table 3). Reasons for not undergoing reconstructive intervention were severe respiratory complications (n = 1), death before reconstructive intervention (n = 1), and data were missing for the reason in 6 patients (16.7%). Twenty-six patients (86.7%, RA n = 13, RS n = 13) underwent surgical reconstruction, of whom 3 (11.5%) developed postoperative complications (i.e. wound dehiscence (n = 1), anastomotic leakage requiring loop ileostomy (n = 1), and neurogenic bladder (n = 1)). Different types of surgical reconstruction were performed, of which PSARP (RA n = 9, 69.2%, and RS n = 6, 40.0%) and surgeries with colo-anal and/or anorectal anastomosis (RA n = 2, 15.4%, and RS n = 2, 13.3%) were performed most often. A complete overview of reconstructive interventions can be found in Table 3.

**Table 3:** Overview on all performed reconstructive interventions

	n (%)
***Rectal atresia, n* = *13***	
*Surgical reconstructive interventions*	
PSARP	9 (69.2)
Rectum resection	
Colorectal anastomosis	1 (7.7)
Anorectal anastomosis	1 (7.7)
Transanal pull-through	1 (7.7)
Opening layer and dilatation under anesthesia	1 (7.7)
***Rectal stenosis, n* = *15***	
*Surgical reconstructive interventions*	
PSARP	
Anterior dentate line sparing procedure	3 (20.0)
Partial rectum resection, with partial ano-cutaneous (dorsal) and colo-anal anastomosis	1 (6.7)
Including excision of sacral teratoma and closure of cerebrospinal fluid cyst	1 (6.7)
Not specified	1 (6.7)
Mini-PSARP	
Anterior dentate line sparing procedure	1 (6.7)
Removal of mass superior (1.5 cm) of the anal verge	1 (6.7)
Modified PSARP	
Only dorsal incision	1 (6.7)
Rectum resection	
Colo-anal anastomosis (anterior hemicircumference) and colo-skin anastomosis (posterior hemicircumference)	2 (13.3)
Anoplasty	
Not specified	1 (6.7)
Transrectal plasty at posterior rectal stenosis	1 (6.7)
*Non-surgical reconstructive interventions*	
Dilatation of stenosis	2 (13.3)

*PSARP* posterior sagittal anorectoplasty. *n* number

### Functional outcomes

**Fig. 1:**
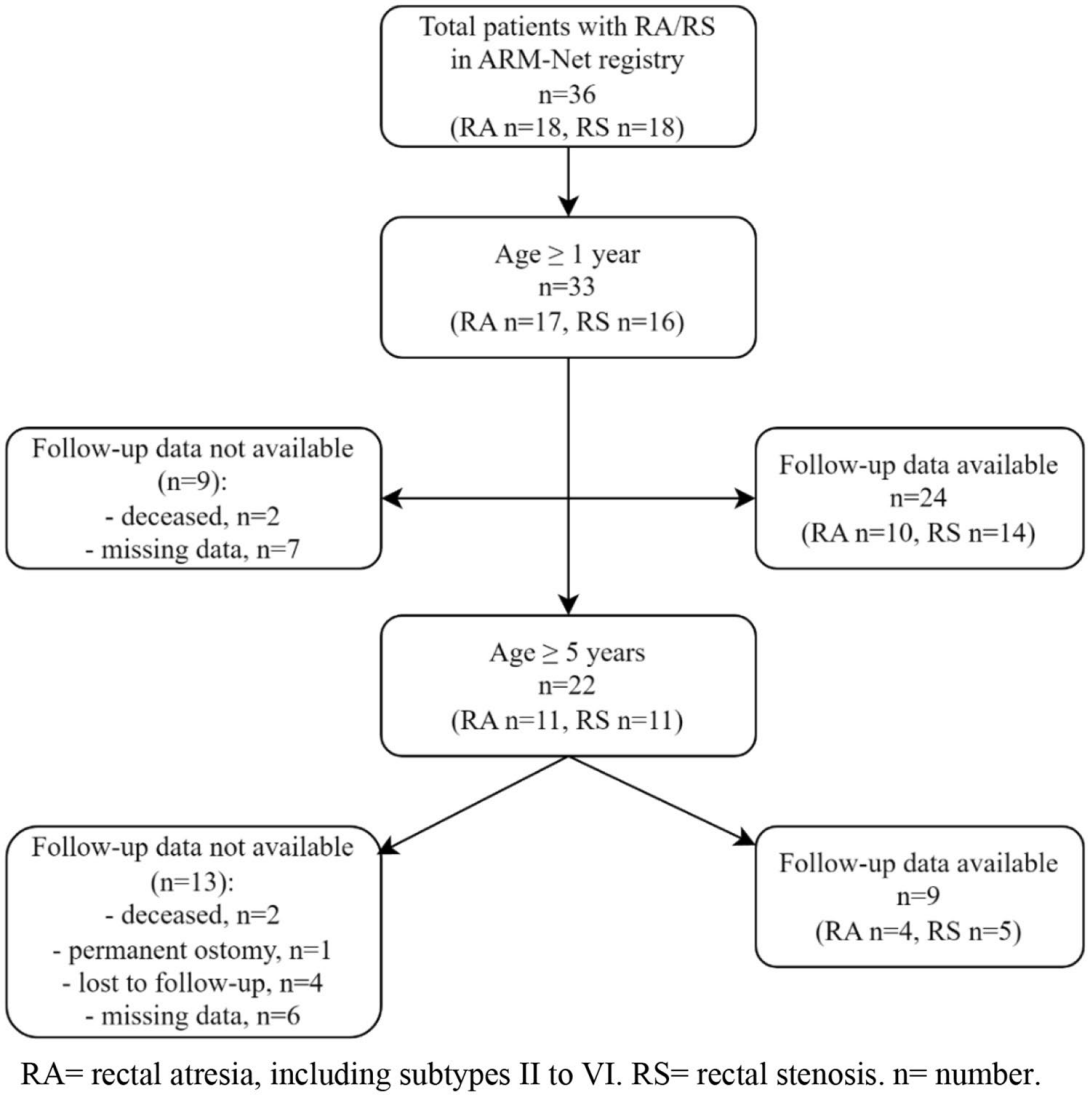
Flowchart on the availability for 1- and 5-year follow-up data on functional outcomes. *RA* rectal atresia, including subtypes II to VI. *RS* rectal stenosis. *n* number

In total, 33 patients were ≥ 1 year of age at analysis (RA n = 17, RS n = 16). One year follow-up data on functional outcomes was not available for 9 of 33 patients (27.3%, RA n = 7, RS n = 2) due to the patient’s death (n = 2) and missing data (n = 7) (Fig. 1). At 1-year follow-up, 11 of 24 patients (45.8%, RA n = 5, RS n = 6) were constipated (i.e. following PSARP (n = 7), anorectal anastomosis (n = 1), transrectal plasty (n = 1), and without surgical intervention (n = 2)). Nine patients (RA n = 4, RS n = 5) required treatment for their constipation, including (a combination of) dietary adjustments (n = 3), stool softeners (n = 5), laxatives (n = 3), or an enema (n = 3). Two patients did not undergo any treatment. Median defecation frequency was 2.0 times a day (IQR 1.0–2.5). At 1-year follow-up, 3 patients were still undergoing dilatation of the neoanus, 15 had completed the therapy, and 5 were never dilated. Of the 18 patients that underwent dilatations, 11 (61.1%) reported pain during dilatations, and 7 (38.9%) never had pain. Data on the presence of dilatations was missing for 10 patients (30.3%).

In total, 22 patients were ≥ 5 years at analysis (RA n = 11, RS n = 11). Five year follow-up data on functional outcomes was not available for 13 of 22 patients (59.1%, RA n = 7, RS n = 6) due to the presence of a permanent ostomy (n = 1), death (n = 2), lost to follow-up (n = 4), and missing data (n = 6), leaving 9 patients with available 5-year follow up data (Fig. 1). At 5-years follow-up, 8 of 9 patients (88.9%, RA n = 4, RS n = 4) were constipated, all requiring treatment with (a combination of) laxatives (n = 6) or an enema (n = 4). Defecation frequencies varied from every other day to twice a day (n = 6), but also frequencies less (n = 2) or more often (n = 1) were reported. Daily soiling was present in 2 patients, whereas 3 patients experienced soiling less than once per week. Most patients had no social problems (n = 6). However, also sometimes social problems (n = 1), problems causing restrictions in social life (n = 1), and severe social and/or psychic problems (n = 1) were mentioned.

## Discussion

This study provides an overview on the patient characteristics of, and treatment strategies performed in, a relative large number of patients with the rare ARM types, RA (n = 18) and RS (n = 18). More than two third of the patients had additional anomalies (n = 23, RA n = 13, RS n = 10). Various treatment strategies were performed, with ostomy in 73% (RA n = 16, RS n = 6) of the patients, and surgical reconstructive treatment in 87% of the patients with RA/RS. PSARP was the most often performed reconstructive surgery for both patients with RA as well as with RS (RA n = 9, RS n = 6). Postoperative complications occurred in 36% of the patients after ostomy surgery and 12% after reconstructive surgery. At 1-year follow-up, 11 of 24 patients (RA n = 5, RS n = 6) were constipated, of whom 9 required therapy. At 5-year follow-up, 8 of 9 patients (RA n = 4, RS n = 4) were constipated, all requiring therapy, but 5-year follow-up data was scarcely available.

The presence of additional anomalies are described for all patients with ARM, regardless of the type of ARM. Also in this study, additional anomalies were identified in 64% of the patients (RA 72.2%, RS 55.6%), which is slightly higher compared to the cohorts including patients with any type of ARM (range 32–62%) [5, 14–16]. Syndromic, cardiac, and skeletal anomalies were the most frequently identified anomalies in our patient population with RA/RS. With multivariable regression analysis, only the presence of syndromes was independently associated with the presence of additional anomalies. Due to the high prevalence of additional anomalies, it is our opinion that screening for these subsequent anomalies is of great importance for patients with RA/RS.

This study showed a wide variety in applied surgical treatment for patients with RA/RS. As described in previous studies, different types of RA/RS have different disease morphology and therefore might require different treatment approaches [3, 8, 17]. Due to the rarity of the disease, and relative low number of patients available, no recommendations can be made on optimal treatment strategy for the different types of RA/RS based on the results of this study. Postoperative complications were only described for 12% of the patients (n = 3) undergoing surgery. Again, due to the heterogeneity in the results, no differentiation could be made on the surgical intervention carrying the least postoperative complications, and, therefore being the safest. However, assessing PSARP and pull-through procedures as the reconstructive interventions most often performed, functional outcomes might differ due to the nature of these interventions. For instance, with PSARP an incision is made in the sphincter complex, resulting in the formation of scar tissue and subsequent potential damage to the surrounding nerve tissue. In contrast, with pull-through procedures, the sphincter mechanism might undergo potential overstretching, resulting in an inadequate closure mechanism. In addition, previous studies often report on short-term postoperative outcomes after (non-)surgical reconstructive intervention for RA/RS (e.g., on different types of complications), whereas literature on mid- and long-term functional outcomes of patients with RA/RS is lacking [8, 11, 18]. In line with this scarcity in available literature, this study showed that follow-up data was not available for 27% of the patients at 1-year, and 59% at 5-years follow-up. At present, in the ARM-Net registry, only 1-year follow-up data is registered and surgeons were contacted additionally to retrieve 5-year follow-up data. Unfortunately, only few surgeons (n = 8) responded to this request. However, it might be of great importance to evaluate these mid- and long-term outcomes, since at 5-year follow-up, 8/9 patients in whom data was available, were constipated and required subsequent bowel management. Additionally, soiling was reported for 5 patients that might eventually negatively influence their quality of life.

In total, 14 patients (38.9%) were born after 2017. However, in this study, despite the proposed classification by Sharma and Gupta in 2017, the type of RA/RS was specified for none of the patients in the ARM-Net registry. Since RA and RS are very rare conditions, uniform reporting and registration is of great importance to enhance generalizability of data reported in different studies. Therefore, future studies should adopt and implement this proposed classification by Sharma and Gupta, taking into account RA/RS as different entities and not to be mistaken by anal stenosis, to improve uniformity, and decrease heterogeneity in the different types of RA/RS. Because of these different types, uniform data representation is also important for improving data repeatability. In order to make this possible, future research should be conducted among international centers or networks to gain a larger cohort of RA/RS patients.

This study should be interpreted in light of some strengths and limitations. First, to our knowledge this study reports the largest number of patients with RA/RS, very rare types of ARM, in current available literature. Second, we were able to show characteristics on these rare patient groups, with the presence of additional anomalies in 72% and 56% of the included patients with RA and RS respectively. Additionally, a large variety in treatment strategies was performed. However, due to the retrospective nature of this study, some limitations occurred. First, information bias is likely to be present. Subsequently, part of the data from the ARM-Net registry are collected prospectively, but the additional questions concerning 5-year follow-up were collected retrospectively. Therefore, some degree of bias, i.e. incorrect classification or exclusion of data, might have occurred. Second, despite n = 18 for both conditions being a relatively large number for RA/RS, objectively few patients could be included, potentially resulting in a wide variety in reconstructive treatment strategies performed. Third, classification as proposed by Sharma and Gupta (2017) was not applied for the majority of patients, and therefore no clear estimation on the distribution of the different RA/RS types could be provided [4]. Finally, 5-year follow-up data was only available for a small number of patients, and therefore no accurate evaluation of long-term functional outcomes could be done.

Even though numbers are small, it is important to report these data, to open a discussion on what optimal treatment is for patients with different types of RA/RS. In order to do so, future research could evaluate different treatment strategies currently performed for the different types of RA/RS, to eventually create a best practice treatment strategy. In addition, it should be investigated whether the identification of additional anomalies prior to primary intervention were of influence in choice of treatment strategy, and if treatment strategies were subsequently altered after identification of these additional anomalies.

In conclusion, RA and RS are very rare types of ARM, representing 1.3% of all patients included in the ARM-Net registry. The majority of patients with RA/RS had additional anomalies, and various treatment strategies were performed in patients with RA/RS. Most patients had an ostomy followed by reconstructive surgical treatment, of which PSARP was most often performed for both conditions. Follow-up data was scarcely available, especially data on 5-year follow-up was lacking for the majority of patients. Therefore, accurate evaluation of long-term functional outcomes remains challenging.

### Supplementary Information

Below is the link to the electronic supplementary material.

Supplementary file1 (DOCX 22 KB)

## Data Availability

Requests for data sharing will be considered by the study steering group upon written request to the corresponding author.
